# Optimising the use of electronic medical records for large scale research in psychiatry

**DOI:** 10.1038/s41398-024-02911-1

**Published:** 2024-06-01

**Authors:** Danielle Newby, Niall Taylor, Dan W. Joyce, Laura M. Winchester

**Affiliations:** 1 Nuffield Department of Orthopaedics, Rheumatology and Musculoskeletal Sciences, Centre for Statistics in Medicine, University of Oxford, Oxford, UK; 2 Department of Psychiatry, University of Oxford, Oxford, UK; 3 Department of Primary Care and Mental Health and Civic Health, Innovation Labs, Institute of Population Health, University of Liverpool, Liverpool, UK

**Keywords:** Psychiatric disorders, Depression, Bipolar disorder

## Abstract

The explosion and abundance of digital data could facilitate large-scale research for psychiatry and mental health. Research using so-called “real world data”—such as electronic medical/health records—can be resource-efficient, facilitate rapid hypothesis generation and testing, complement existing evidence (e.g. from trials and evidence-synthesis) and may enable a route to translate evidence into clinically effective, outcomes-driven care for patient populations that may be under-represented. However, the interpretation and processing of real-world data sources is complex because the clinically important ‘signal’ is often contained in both structured and unstructured (narrative or “free-text”) data. Techniques for extracting meaningful information (signal) from unstructured text exist and have advanced the re-use of routinely collected clinical data, but these techniques require cautious evaluation. In this paper, we survey the opportunities, risks and progress made in the use of electronic medical record (real-world) data for psychiatric research.

## Introduction

Psychiatry covers a vast heterogeneous group of mental disorders, manifesting as unusual mental or behavioural patterns that can impact an individual. Psychiatric research has increased rapidly to help in understanding the mechanisms of disease and treatments of multiple mental health and neurological disorders. With the growth of large-scale data, such as electronic medical records (EMR), research into psychiatric disorders can benefit from this and can provide multiple opportunities in psychiatric research that will produce evidence that could be incorporated into standards and guidelines. This, in turn, will directly impact clinical decision-making and, ultimately, the patient benefit.

Electronic medical (health) records (EMR) contain data describing clinical interactions, administrative, medico-legal, diagnostic, intervention, prescribing and investigations collected for the purposes of providing routine clinical care. In psychiatry (unlike other medical specialities), detailed clinical data is most often in unstructured, narrative “free text” and depending on the healthcare system, other clinical data (e.g. structured data recording the results of investigations and prescribing) will be available to varying degrees. Rather than representing unadulterated “real world” data, the potential for EMRs to provide relevant, reliable and rich data varies depending on the application; for example, reusing EMR data for predicting child and adolescent mental health problems after first contact with services [1] demonstrated limited utility. An often unrecognised problem with EMR data—particularly as a source of observational, retrospective cohort data—is that the content reflects treatment as usual (i.e. extracted prescribing data will likely display indication biases), the culture of the institution and its practitioners (e.g. unstructured narrative data might reflect the mixing of administrative, medico-legal and clinical data) and the institution’s implementation of an EMR platform [2, 3] (for example, whether the pathology EMR system in use at the same hospital are linked meaningfully to the central EMR being used for research data extraction) [4].

There is still a common consensus that randomised control trials (RCTs) are the gold standard to provide causal evidence for the efficacy, effectiveness and benefits of interventions, and for inferential modelling of risk factors for mental illness. However, RCTs can be expensive, time-consuming, unethical to conduct and generally have short follow-up times compared to observational studies. Some argue that this delivers evidence lacking generalisability to patients and their presentations in routine clinical care and excludes those patients whose risk formulation excludes them from clinical trials. Therefore, evidence derived from EMR-based research has the potential to complement evidence from controlled trials, especially when considering health equity and reproducibility [5–7]. Furthermore, causal inference methods are being introduced to address some of the biases in observational research using EMR data.

This narrative review concentrates on how research on neuropsychiatric disorders (such as depression, bipolar, schizophrenia, anxiety, eating disorders and dementia) can utilise big data such as EMRs to generate evidence to inform clinical decision-making and, importantly, improve patient outcomes. Examples of types of data that can be utilised, examples of use, and the benefits and limitations of EMR will be discussed. Finally, a summary of how EMR can be further advanced, such as the use of genetics and data triangulation will be discussed to further help optimise EMR for psychiatry research.

## Data sources for large-scale psychiatric research

**Fig. 1:**
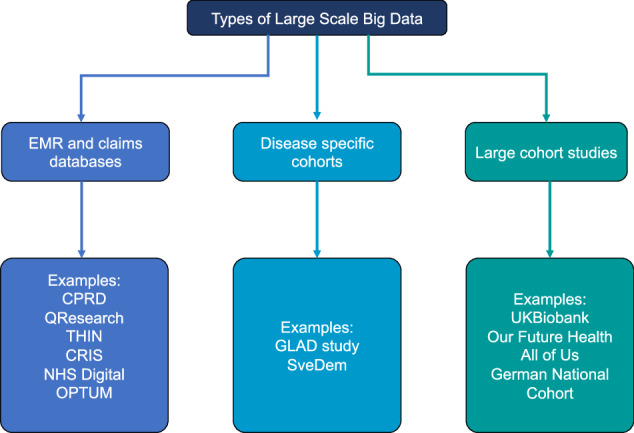
Examples of potential data sources for psychiatric research. CPRD: Clinical Practice Research Datalink [174], QResearch (https://www.qresearch.org/), THIN: The health improvement network (https://www.the-health-improvement-network.com/), CRIS: Clinical Record Interactive Search, OPTUM (https://www.optum.com/), NHS Digital (https://digital.nhs.uk/), GLAD study: Genetic Links to Anxiety and Depression Study [175], SveDem: The Swedish Dementia Registry [176], UK Biobank [177], Our Future Health (https://ourfuturehealth.org.uk/), All of Us (https://allofus.nih.gov/), German National Cohort [178]. EMR and claims databases contain a variety of data formats which can be classified as structured or unstructured [69]. Structured data includes information such as age and gender, measurements such as blood pressure readings, height and also diagnosis codes, laboratory tests and medication prescribing. Whereas unstructured text includes narrative data such as clinical notes (e.g. biopsychosocial formulations, differential diagnoses, mental state examinations and risk formulations). Compared to narrative, unstructured data, structured data is easier to process with little pre-processing because it is stored in a standardised format. EHR and claims databases have vast patient numbers covering all diseases and disorders, giving the opportunity to look at psychiatric conditions and their comorbid diseases.

There is a vast variety of data sources that can be used for large-scale research in psychiatry. Before designing a study, it is important to understand different data sources and their strengths and limitations to ensure a research question can firstly be answered and then without significant biases. Broadly speaking, large-scale data resources can be roughly grouped into three types (Fig. 1).

Disease registries contain patients with a specific condition and collect patient information longitudinally [8]. As the early and accurate diagnosis of psychiatric conditions is essential for better disease monitoring and management, registries represent a valuable tool for studying the known risk factors, as well as identifying new risk factors and markers that may help improve the accuracy of diagnostic procedures in psychiatry [8]. Disease registries also allow insights into medication use and their effectiveness and adverse effects in managing mental health conditions. Therefore, disease registries play an important part in improving health outcomes for patients and reducing healthcare costs [9].

Large population cohort studies contain large sample sizes and extensive phenotypic, imaging and biological measurements, including genetics [10]. Due to the large number of participants, this allows researchers to investigate psychiatric conditions with sufficient statistical power. With genotyping carried out for these large cohort studies this allows for the complex relationship of multiple small-effect genetic and environmental influences of psychiatric conditions to be studied [11]. One of the caveats of some large population studies is the potential lack of representativeness [12] and diversity, particularly for those with mental health conditions [13]. Other data sources that are potentially important for psychiatry research include data collected from wearables, mobile phones and social media platforms [14–18].

## The current uses of EMR to enhance psychiatry research

EMR is used to generate a wide variety of evidence to inform and improve patient care ranging from using curated EMR data for epidemiology to identifying novel risk factors, opportunities for innovation in treatments and predictive analytics for those at risk and/or treatment response. The main uses related to the psychiatry field are discussed below.

### Comparative effectiveness studies

Comparative effectiveness research using EMR can provide evidence to improve patient care and reduce healthcare costs. This is done by comparing the benefits and harms of alternative treatments or methods to prevent, diagnose, and treat a variety of health conditions [19, 20]. There are a variety of study designs that can be implemented to understand the effects of different mental health disorders, such as anxiety and depression, on quality of life before and after diagnosis [21], as well as the effectiveness of different medications [22–24] and different treatment regimes [25] for a variety of mental illnesses [26–28]. For neurodegenerative conditions such as dementia, there is also a growing body of evidence using EMR to investigate the potential benefits and harms of licensed medications [29–34]. As there is evidence that common diseases such as diabetes and hypertension are probable risk factors [35], this suggests that treatments for these conditions may influence cognitive decline and potentially modify dementia risk. On the other hand other anticholinergic medications [36, 37] and benzodiazepines [38, 39], may accelerate decline or increase the risk of dementia.

### Descriptive studies

Descriptive studies quantify features of the health of a population of interest. This leads to knowledge that could generate hypotheses for aetiologic research and inform action in the population it concerns [40, 41]. The use of descriptive studies can be used to estimate the burden of disease in a population at a certain point in time or over time (e.g. incidence and prevalence). For psychiatry, descriptive studies can be used to ascertain if there have been changes in trends of mental health disorders such as depression [42] and anxiety [43] as they present to healthcare services or within certain populations of patients with chronic diseases, mental health conditions [44] and life-limiting diseases such as cancer [45]. This can help develop strategies that could mitigate and treat those with mental health conditions and descriptive epidemiology has been vital to understanding the impact of the COVID-19 pandemic on mental health [46–48]. Other types of descriptive studies entail describing drug utilisation and adverse drug reactions to medications [49, 50]. These studies can provide information regarding potential over-, under- or mis-prescribing of medications leading to poorer patient outcomes, particularly in high-risk populations such as those with mental health or neurological conditions [49, 51, 52].

### Prediction modelling

Predictive modelling attempts to complement evidence-based medical practice by providing methods for using clinical data to estimate an individual’s probability of, e.g. experiencing benefit or harm from a treatment, experiencing an outcome (prognosis) or having a diagnosis [53]. A critical stage in developing predictive models is external validation and calibration of a tentative model, ideally in a prospective evaluation. EMRs are often conceived as ideal data sources for predictive model development and, sometimes, validation; but currently, there is limited evidence for the robustness of predictive models in psychiatric applications more generally, for example, in a systematic review of risk prediction models [54], of 89 studies, only 29 had been subjected to external validation and 1 study was considered for implementation.

Common clinical domains for predictive modelling include suicide risk [55], diagnostic trajectories [56], treatment outcomes in depression [57] and identification of dementia cases [58]. Notably, many well-designed and implemented models (e.g. those with robust validation) have tended to use national registry data (rather than EMR-derived data). Whilst individual studies using EMR data have shown promise [59–61], there is little synthesised evidence demonstrating the value of EMRs for predictive modelling. Registry data is (importantly) different from EMR data (even if one federates a number of organisation’s individual EMRs) because registries are samples of the whole population, whereas EMRs are selection-biassed (i.e. only people who are unwell and require input from services will be visible in EMRs).

## Challenges and opportunities with using electronic medical records for large-scale psychiatry research

One of the most important considerations when utilising EMR for research is that it is collected for healthcare and not for research purposes. It is important to understand this when using EMRs for research because they contain a vast amount of data that reflects medico-legal and administrative concerns, rather than being clinically relevant.

### The Big Data Paradox

Big data can be characterised by its variety, volume, velocity, and veracity [62, 63]. In context, EMR can be considered “big data” (due to its variety, volume and veracity) containing information in the order of thousands to millions of patients. The large number of patients and coverage of clinical conditions allow opportunities to study rare events or disorders (i.e. exploiting volume, variety and veracity) encountered in “real-world” clinical practice [64]. However, EMR is collected to support healthcare delivery and services, which gives rise to heterogeneity in the data collected. The volume of EMR datasets promises large sample sizes but this often leads to an assumption that derived error and uncertainty estimates will be necessarily more precise. However, this commonly received wisdom does not always hold; the “big data paradox” [65, 66] describes how increasing the sample size *alone* does not guarantee a more precise estimate of e.g. sample averages. In studies of survey data, vaccine uptake and the prediction and tracking of flu [67, 68], large sample sizes yielded misleadingly narrow uncertainty estimates leading to biased population inferences. We should be mindful of the quality, heterogeneity, and problem difficulty that are all functions of the data used, how it is collected, and the specific application or re-use of that data [65].

### The dominance of unstructured text in electronic medical records in psychiatry

Unstructured data, such as free text, requires considerable pre-processing and, usually, domain expertise and human annotation. A major problem with clinical free text is the language used by clinicians is often idiosyncratic, with frequent abbreviations (sometimes, with parochial meaning such as the names of clinical services), and varied medical vocabularies [69]. Drug names, for example, often have different brand names in different national territories or “class” nomenclature (i.e. “antidepressant”) depending on the institution, requiring ontologies to be developed for mapping between synonymous terms (e.g. the Unified Medical Language System [70]) to assist pre-processing before being used in analyses or model development. Within psychiatry and mental health the number of clinical notes for any individual can be very large and written in a narrative but terminologically dense manner and often contain a high proportion of redundant text [71]. Further, unlike other medical and surgical specialities (that can utilise EMR-based sources of routinely collected structured data), psychiatry is far more reliant on clinical information such as symptoms, behaviour and clinical assessments within the unstructured notes. The major task is to represent this clinical text in a useful way for both algorithms and clinicians alike.

The computational processing and analysis of human language found in the unstructured text (clinical notes) falls under the broad field of natural language processing (NLP), which pertains to the statistical [72, 73] and deterministic (e.g. rule-based) representation and processing of language. NLP seeks to represent words, sentences, paragraphs and sometimes, the entire text corpus in such a way that algorithms can be deployed to automate task-specific analyses of the text. Contemporary NLP usually combines rule-based methods with statistical (usually machine and deep learning methods) to represent written and spoken language. The current state of the art for NLP focuses on pre-trained language models (PLMs, very-large deep learning NLP networks trained using a language modelling objective) like BERT [74] and GPT-3 [75]. PLMs better capture semantic nuances contained in sequences of text and have seen state-of-the-art performance in a considerable number of domains e.g. finance, internet of things, biomedical [75–77]. Most impactful applications of PLMs to EMR-free text have focused on Information Extraction, e.g. named entity recognition. This has spawned a number of tools to create structured representations of that free text to aid clinical decision support, such as MedCAT [78], NeuroBlu [3], and Med-7 [79]. However, the research into the representation of clinical notes in psychiatry as a whole is still relatively limited, especially in relation to the latest trends in NLP.

A concrete example of a challenge in NLP applied to narrative EMR data in psychiatry concerns the vernacular use of diagnostic terminology; for example, a healthcare professional might summarily describe their impression that “the patient seems depressed”. In isolation, this statement might refer to signs (observations by the professional), symptoms (difficulties reported by the patient) or a summary diagnosis (the signs and symptoms observed in this clinical encounter meet diagnostic criteria for a depressive disorder or episode). Similarly, a recording of clinical *state* might read “Mood: normal” and could refer to the patient’s mood being *normal for them* (referencing a previously observed clinical state), a *normative* assessment representing a lack of pathology (where the clinician’s recording references their own experience of the population of people with “abnormal” mood) or could represent a change over time (i.e. that the patient’s mood has returned to some baseline). Resolving these different interpretations remains difficult using data-driven lexical or statistical analyses of language and necessarily, resource-intensive expert human annotation is required.

### Resource challenges using machine learning-based NLP within psychiatry

Contemporary neural networks (NN) are computationally expensive when compared to other mature machine- and statistical-learning methods. Practical development of NN models requires parallel processing using Graphical Processing Units (GPU) that are costly. The last few years have seen neural networks reaching the size of hundreds of billions of parameters, and the amount of data used to train them is usually comparably vast. A prominent large language model, GPT-3 [75], has ~175 billion model parameters (by comparison, the human brain has ~86 billion neurons). Commercial interests often obfuscate accurate costing, but speculative estimates are of the order of several million US dollars to train models of this magnitude [80, 81]. This trend of increasing performance through scaling of model size/complexity is problematic for resource-constrained environments such as publicly-funded hospitals (i.e. the UK’s National Health Service).

A crucial component of any AI/ML-driven algorithm or tool is that it is trusted and usable by human clinicians and patients; Critically, imbuing trust in a model requires that the algorithm deliver outputs that include justifications or reasons for reaching a given output or decision, sometimes referred to as XAI (eXplainable AI). Many ML methods (and especially deep learning neural networks) are opaque or “black-box” models, where the computational processes that intervene between input and output are too complex to be easily understood by any human user. There is an active research field dedicated to illuminating the machinery of such models, although the concept of what constitutes an *explainable* or *interpretable* model remains controversial [82]. If clinicians and patients are to trust an AI/ML model, they will likely favour model transparency and simplicity—often described as intrinsically interpretable models [83]—over the often modest performance gains given by complex DL models [84]. Free text data in sensitive (and, for psychiatry, often stigmatising) settings carries serious privacy risks due to the difficulty in adequately anonymising data and removing personally identifiable content [69]. For this reason, these data are often warehoused with strict data access regulations that necessarily inhibit reproducibility and replicability efforts.

### Problems with data linkages and selection bias in EMR

Linking together information about the same individual across multiple data sources can further enhance existing data [85, 86], improve the quality of information, and offer a relatively quick and low-cost means to exploit existing data sources. One benefit of data linkage in psychiatry is it can provide additional information on other non-psychiatric conditions and medications [87], allowing more detailed information about patient’s medical history, which can be used to reduce biases in research studies. Although data linkages can improve knowledge about psychiatric research [87, 88], there are limitations. Errors in the data linkage process can introduce bias of unknown size and direction, which could feed through into final research results, leading to overestimating or underestimating results [89]. Missingness of different participant characteristics in EMR, such as age, gender and race, can also lead to systematic bias and issues with the validity and generalisability of research results [90, 91].

Selection bias is a common problem in observational research and occurs when characteristics influence whether a person is included in a group. For example, in psychiatry, only those with extreme mental health conditions enter secondary care due to the different priorities of healthcare providers and government funds. Therefore, any research studies using EMR in secondary care will differ from the general population [92]. Furthermore, selection biases can exacerbate existing disparities, such as those relating to ethnicity, sexual orientation and socioeconomic status, that can lead to inequalities in treatment and healthcare [93–97]. Findings from psychiatric research conducted in selected groups should be interpreted with great caution unless selection bias has been explicitly addressed.

## Phenotyping in psychiatric research

Phenotyping is the process of identifying specific patients with a clinical condition or characteristic(s) based on information in their EMR [98]. It can involve combining different types of data such as diagnosis codes, procedures, medication data, laboratory and test results, and unstructured text [99] with growing interest in using data from smartphones and other digital wearables [15, 100]. Phenotypes can be derived using algorithms that use filters and rule-based algorithms or machine learning methods based on structured data [101, 102]. The Electronic MEdical Records and GEnomics (eMERGE) Network [103] and CALIBER [104] have both shown that phenotypes can be identified and validated and consequently used in research. Patients identified with a specific phenotype can be included in cohort studies in order for further study of risk factors or drug safety surveillance, genetic studies as well as recruitment for clinical trials [105–110]. The psychiatry field presents a unique challenge for phenotyping as the majority of psychiatric diagnoses typically rely on self-reported symptoms, behaviour and clinical judgement, meaning a combination of structured and unstructured text has been shown to give rise to more accurate phenotypes with less misclassification of cases [111, 112]. Problems arise with phenotyping when there is no consistency in the phenotyping process, only using structured data may not accurately represent the disease status of the patient, what types or combinations of data could be used from different healthcare datasets and the lack of translation of phenotypes to different health care settings and countries [113]. As phenotyping is a dynamic process, it requires clinical expertise and multiple cycles of review and can take many months of development [114]. Once a phenotype has been derived, validation of the phenotype is a critical process [115]. A phenotype must have high sensitivity and specificity, limiting both false positives and false negatives. Validation can be done using a variety of different approaches [104], such as by cross-referencing different data EMR sources and case note reviews by clinical experts to confirm a diagnosis based on the phenotype developed. Accuracy measures can then dictate how useful a phenotype will be for use in further research [106, 116, 117].

## Future considerations for optimising the use of electronic medical records in psychiatry

There are many examples of the use of EMR to generate evidence in psychiatric research. However, to aid in the improvement and research applications of EMR we discuss future considerations which could optimise the use of EMR.

### Design, statistical techniques to address biases and reporting in observational psychiatric research

The design, analysis and reporting are vital components for optimising the use of EMR. Observational research using EMR is utilised because RCTs that would answer causal questions are sometimes not feasible, unethical and take too long. By applying the study design principles of RCTs to observational studies, the causal effect of an intervention [118, 119] can be estimated, and this helps avoid biases such as selection and immortal time biases [120]. This approach, called “target trial emulation” [118], uses EMR to emulate a clinical trial—the target trial—that would answer the causal research question. If target trial emulation is successful the results from observational data can yield similar results to the RCT [121–125]. Target trial emulation is now being used for a wide variety of conditions, such as showing potential beneficial effects of statins with dementia risk [126] and harmful effects of protein pump inhibitors with dementia risk [127]. Other applications include determining optimal drug plasma concentrations in bipolar disorder [128] and establishing the risk of diabetes with anticonvulsant mood stabilisers [129]. Target trial emulation cannot remove bias due to the lack of randomisation of observational data [118]. However, methods to address this, such as propensity scores, can be applied to reduce this confounding [130, 131].

Clinical decision support tools for psychiatry could include identifying or detecting those at risk of certain disorders, illness progression/prognosis and using treatment response data to improve personalised care. However, specifically for predictive models, research has shown that over 90% were at high risk of bias [53]. Therefore, in order to optimise the use of EMR for developing clinical decision support, we require careful attention to model development, sample sizes [132], internal and external validation, including calibration and assessment of clinical utility and generalisability should be adopted [59, 133].

Studies using EMR can be prone to publication bias and reporting bias [134, 135]. On top of this, published research often omits important information or the information is unclear and very often, the nature of EMR data means it cannot be shared for interrogation, reproducibility and replication studies. These biases are a concern because they undermine the validity of studies. Study analysis plans and study results should be reported transparently, including what was planned, what was carried out, what was found, and what conclusions were drawn. Researchers can now register statistical analysis plans for a study prior to analysis (e.g. clinicaltrials.gov, researchregistry.com, encepp.eu) and the STROBE [136] and TRIPOD [137] guidelines offer a checklist of items that should be addressed in articles reporting studies to increase transparency [138, 139]. Furthermore, in order to improve reproducibility and ambiguity, analytical code should also be freely available [140].

### Precision medicine to provide individualised healthcare

With the increase in the availability of accurate deep phenotyping information from unstructured text researchers will be able to make more precise insights about disease outcomes from clinical information. This has expanded the scope of evidence-based prediction and tools designed to triangulate evidence from multiple sources are now being developed for applications in precision medicine. For example, the Petrushka [105] web-based tool uses data from multiple sources, including QResearch (primary care), EMR (secondary care) and available literature to make personalised medication recommendations in individuals with unipolar depression. Other projects seek to incorporate other data modalities, such as wearables to give a more detailed digital phenotype [141]. However, further validation is needed to convince clinicians of the benefits of supported clinical decision-making.

### Triangulation of evidence from multimodal data for large-scale psychiatry research

There is a vast array of data acquired in research and healthcare which covers a variety of different modalities. These different modalities, such as omics, histology, imaging, clinical and smart technology, can help researchers unveil novel mechanistic insights to help understand crucial information about the complexity of mental health and neurological conditions. Triangulation of evidence is an approach where one can obtain more confidence in results by carrying out analyses integrating different statistical methodologies and/or data modalities [142, 143]. The key is that each analysis has different sources of potential bias that may be unrelated to each other. If the results from each different analysis point to the same conclusion, this strengthens the confidence in the findings obtained. Examples of this triangulation approach in mental health research include assessing the relationship between cultural engagement and depression, where the authors used three different statistical methodologies with different strengths and weaknesses to show lower cultural engagement is associated with depression outcomes [144]. Other examples used observational data and genetic data to triangulate evidence between smoking and suicide ideation and attempts [145], and anxiety disorders and anorexia nervosa [146]; however, the triangulated results were inconsistent with each other, potentially questioning the causal relationships established using any of the sources. For psychiatry, triangulation could be used by applying different statistical approaches, using different EMRs across different countries and healthcare settings and/or integrating other non-EMR data as discussed below to help provide further understanding regarding causality and optimise big data in psychiatric research.

### Incorporating biomarkers in EMR phenotyping

Biomarkers are biological measures utilised to better diagnose, track or predict psychiatric disorders. These can range from clinical assays and brain imaging to digital biomarkers from wearables. In EMR research, they can be used to help define disease phenotypes or better understand outcomes and applications in precision medicine. In Alzheimer’s Disease, fluid biomarkers (cerebrospinal fluid and blood plasma) for the tau protein are used to determine disease pathology to aid in trial recruitment. Biomarkers of neurodegeneration have been successful, neurofilament light measured in blood or CSF can be used to assess axon damage [147]. Measures of inflammation, such as C-reactive protein, have applications in many psychiatric disorders. Disorder-specific markers have been identified and replicated in meta-analyses for Vitamin B6 in schizophrenia and basal cortisol awakening in bipolar disorder [148]. However, in many psychiatric disorders, translation to clinical applications is limited [148, 149] and further work will be necessary to validate these potential candidates in suitable cohorts.

The suitability of the marker modality should be considered when selecting a biomarker. In mental health conditions, the development of a digital marker captured by remote monitoring might aid in diagnosis by adding information to the self-reporting of symptoms from a patient, for example, if the marker can act as a proxy for behavioural signs of mental illness that cannot be captured by a single measurement of clinical state when consulting a clinician. The development of phone-based applications allows clinicians to collect data on changes through a series of symptom-based questions [150]. However, the future of biomarker discovery is likely the ability to measure, compare and combine multiple variables and here, resources are key. The Penn Medicine Biobank [151] includes genetics and biomarkers alongside EMR to enable precision medicine and the discovery of new phenotypes.

Interrogation of EMRs has revealed the potential value of routinely recorded data to identify and validate the use of existing and exploratory biomarkers. For example, in a study of sepsis, biomarkers were used alongside EMR to study progression [152]. Biomarkers were employed to study different time periods whereby early-life mental health impacts midlife using a panel of markers [153]. Elsewhere the combination of biomarkers and EMR has been recommended for aiding risk reduction profiles and identifying new clinical biomarkers [154].

### The use of genetics for large-scale psychiatry research

The observational nature of many findings precludes drawing firm conclusions about causality due to residual confounding and reverse causation. With the recent explosion of large-scale genetic data available, methods using this data, such as polygenetic risk scores (PRS) and Mendelian randomisation (MR) allow the elucidation of causal relationships between psychiatric disorders, risk factors and drug treatments. Leveraging genetic data using PRS and MR offers a cost-effective approach with the future potential to embed genetic data into healthcare settings to help improve patient care [155] and could provide complementary evidence as part of the triangulation process.

PRS are weighted sums of genetic variants associated with a particular condition [156]. Therefore, PRS can estimate the genetic risk of an individual for a disease or trait. Due to the complex polygenic nature of many conditions (including mental health and neurological conditions [157–161], PRS can only capture a fraction of the overall risk, with clinical and demographic factors usually explaining most variance. This means that on their own, PRS is unlikely to definitively predict future diagnoses in a healthcare setting [162, 163]. However, PRS could be included with other measures to predict future risk and may show promise in aiding clinical decision-making. Adding PRS to risk prediction models alongside other clinical factors such as age, gender and family history has been shown to improve model performance for predicting the risk of conditions such as dementia [164] and certain mental health conditions [162, 165, 166]. On top of this, PRS may have some potential to inform treatment response if polygenetic complex traits can be predicted from an individual’s genetics [167] and those traits are robustly associated with treatment response. PRS can be used in conjunction with other methods, such as Mendelian randomisation [168], to uncover casual insights between complex psychiatric traits and treatments.

**Fig. 2:**
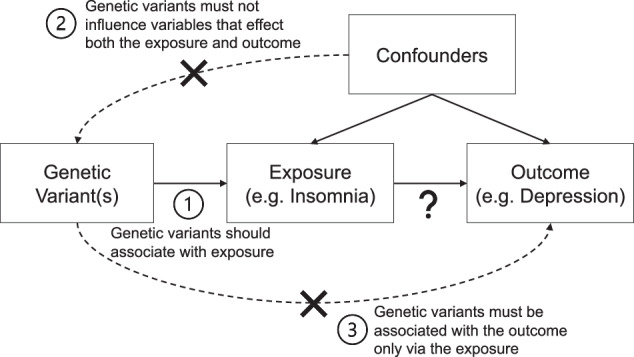
The concept of Mendelian randomisation (MR) and its assumptions. There are a growing number of MR studies being published that show causal effects related to disease-disease associations and drug-disease associations for mental health and old age psychiatry [179–186] with extensions to traditional MR approaches, which could offer further insights [187–189].

Mendelian randomisation (MR) is a statistical approach that uses genetic variants to provide evidence that an exposure has a causal effect on an outcome [169–173] (Fig. 2). A genetic variant (or variants) is used which is associated with an exposure (e.g., insomnia) but not associated with any other risk factors which affect the outcome (e.g., depression). By doing so, any association of the genetic variant(s) with the outcome must act via the variants’ association with the exposure and imply a causal effect of the exposure on the outcome. As genetic variants are inherited randomly at conception, genetic variants are not susceptible to reverse causation and confounding, like observational studies using EMR. Results from MR can help support results from EMR by using data triangulation, as discussed previously.

## Conclusion

Large-scale research approaches are at the forefront of EMR use in psychiatry. With the advances in interpretation using NLP and access to diverse data resources, the scope of research questions is rapidly expanding. However, care is needed to make sure that potential biases are considered. Not considering limitations with big data can lead to incorrect inferences about a population which could mean poorer care for high-risk populations such as those with mental health conditions and neurodegenerative conditions.

In order to optimise EMR for psychiatry a clear understanding of such biases in the data is vital. A researcher must carefully consider if the research question can be answered in the data source they want to use and develop the best study design and statistical analysis. By cautiously incorporating the strengths of the EMR format it will be possible to make exciting contributions to mental health and neurological research.
